# Proteomic analysis of *Clostridium acetobutylicum* in butanol production from lignocellulosic biomass

**DOI:** 10.1186/1753-6561-5-S7-P176

**Published:** 2011-09-13

**Authors:** Kumaran Sivagnanam, Vijaya G S Raghavan, Manesh Shah, Nathan C Verberkmoes, Robert L Hettich, Mark G Lefsrud

**Affiliations:** 1 McGill University, USA; 2 Oak Ridge National Laboratory, USA

## Background

Plant biomass is an abundant and renewable source of energy rich carbohydrates that can be efficiently converted by microbes into biofuels [1]. Butanol is considered as a second generation biofuel when it is produced from lignocellulosic biomass comprising of agricultural and garden wastes that does not compete with the food supplies [2]. *Clostridium acetobutylicum* is a gram positive, spore forming, obligately anaerobic bacteria capable of converting different sugars from lignocellulosic biomass to butanol through acetone – butanol – ethanol (ABE) fermentation process [3]. However, the production of butanol from ABE fermentation process is not economically viable and studies have been performed to understand the utilization of lignocellulosic biomass and regulation of butanol production to improve butanol productivity [4]. Successful industrial butanol production process through ABE fermentation requires complete understanding of the *C .acetobutylicum*. Shotgun proteomics provides a direct approach to study the whole proteome of an organism at molecular level in depth. Therefore, this paper focuses on shotgun proteomic profiling of *C. acetobutylicum* ATCC 824 from butanol fermentation process, elucidating the molecular functional mechanisms of *C.acetobutylicum* in butanol production.

## Materials and methods

The microorganism *C.acetobutylicum* bacterial strain ATCC-824 was cultured [5]and ABE fermentations were carried out in batch mode using glucose substrate which is the most abundant compound present in lignocellulosic biomass. Samples of 10ml were harvested at late exponential phase from the start of the inoculation and proteins were extracted followed by digestion to peptides. The complex peptide solution was desalted through C18solid-phase extraction, concentrated, filtered and for each LC-MS/MS analysis, ~1/4 of the total sample was used based on the protocol used by [6]. Samples were analyzed in technical duplicates through a 2D nano-LC MS/MS system with a split-phase column (RP-SCX-RP) with 12hr runs [7-9]. All MS/MS spectra were searched with the SEQUEST algorithm [10]against *C .acetobutylicum* Uniprot proteome databases [11] and filtered with DTASelect/Contrast [9]at the peptide level. Only proteins identified with two fully tryptic peptides from a 12hr run were considered for further biological study.

## Results

A total of 479 proteins were identified in the proteome analysis of *C. acetobutylicum* from a single data point during the ABE fermentation process and 372 proteins were found to be present in both the first and second MS runs and identified as common proteins. This analysis confirms that 12 proteins were involved in the butanoate metabolism and about 32 uncharacterized proteins were found to be present during the ABE fermentation using glucose substrate. Overall, this is the first study which represents an extensive survey of whole proteome analysis of *C.acetobutylicum* from a single data point by multidimensional protein identification technology (MudPIT) and provides a valuable dataset of *C.acetobutylicum* proteins for a better understanding of the butanol production.
